# Spontaneous tonsillar hemorrhage managed with emergency tonsillectomy in a 21-year-old man: a case report

**DOI:** 10.1186/1752-1947-7-192

**Published:** 2013-07-26

**Authors:** Petros V Vlastarakos, Emily Iacovou

**Affiliations:** 1 ENT Department, Lister Hospital, Coreys Mill Lane, Stevenage, Hertfordshire SG1 4AB, UK; 2 ENT Department, General Hospital of Larnaca, United States of America Avenue, Larnaca, Cyprus

**Keywords:** Bleeding, Hemorrhage, Spontaneous, Tonsil

## Abstract

**Introduction:**

Spontaneous tonsillar hemorrhage is defined as continuous bleeding for more than one hour, or more than 250mL of blood loss regardless of the duration of bleeding. It is an often under-diagnosed and under-reported complication of acute or chronic tonsillitis, with controversial management. We suggest that an emergency tonsillectomy should be performed as first-line treatment for this potentially life-threatening condition on the basis of the relevant anatomy.

**Case presentation:**

A 21-year-old Caucasian British man was referred to the ear, nose and throat emergency service at our facility because of profuse tonsillar hemorrhage, with no history of tonsillectomy. Our patient had been experiencing right-sided swallowing discomfort for five days. On examination, blood spurting from the body of the right tonsil was seen, which was not manageable conservatively. Our patient was taken to an operating theatre, with his pre-operative hemoglobin having dropped by three units within three hours. The bleeding was not controlled by superficial cautery using bipolar diathermy, and a right tonsillectomy with meticulous hemostasis was performed. Our patient was discharged the next day. The histology of the excised tonsil was suggestive of a benign non-specific ulcer, on a background of chronic non-specific tonsillitis.

**Conclusions:**

The tonsillar blood supply comes from branches essentially approaching the tonsil from underneath its body. Ear, nose and throat surgeons and accident and emergency doctors need to be aware that an episode of spontaneous tonsillar hemorrhage is not likely to be controlled conservatively, because the source of bleeding requires removal of the tonsil to be accessed. Hence, performing a tonsillectomy seems a reasonable first-line treatment in such cases.

## Introduction

Spontaneous tonsillar hemorrhage is a rare and often under-diagnosed complication of acute or chronic tonsillitis in the antibiotic era [1], which has been reported in 55 patients in the worldwide literature to date [2]. Griffies *et al*. defined spontaneous tonsillar hemorrhage as continuous bleeding for more than one hour, or loss of more than 250mL of blood regardless of the duration of bleeding [3]. Reported cases indicate an increased incidence in younger patients, associated with a higher mortality rate [2].

Here, we present a case of spontaneous tonsillar hemorrhage and discuss the management of this potentially life-threatening situation.

## Case presentation

A 21-year-old Caucasian British man with tonsillar hemorrhage was urgently referred to our hospital for specialist treatment from a nearby hospital with no out-of-hours ear, nose and throat (ENT) coverage. Despite the ENT senior house officer (SHO) at our hospital having reservations about the apparent presentation, the referring surgical SHO was adamant that the bleeding, albeit not having followed any throat surgery, was profuse.

Our patient’s medical history was largely unremarkable, apart from the recreational use of cocaine five days prior to presentation. Our patient had been experiencing swallowing discomfort for five days, exclusively on the right side of his throat, and mainly at night. He had noticed pus in the right tonsil on the previous morning, and had been prescribed penicillin V by his general practitioner.

Our patient’s hemoglobin during the initial assessment was 13.5g/dL. Our patient arrived by ambulance two hours after his initial referral, around midnight. On examination, the ENT SHO noticed blood spurting from the body of the right tonsil and immediately contacted the on-call ENT surgeon. The on-call surgeon suggested pressure with adrenaline-soaked pledgets, and notification of the on-call anesthetist and the emergency theatre team pending his arrival.

**Figure 1:**
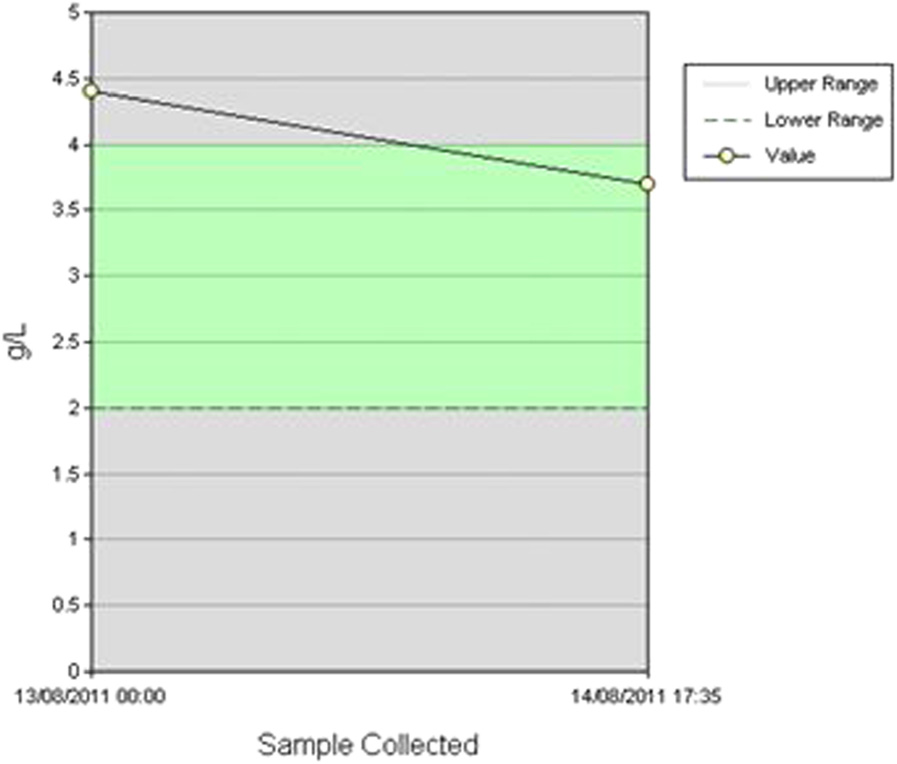
Abnormal initial fibrinogen value as a result of the continuous bleeding of our patient.

Upon his arrival 20 minutes later the ENT surgeon noticed trickling of blood from the right tonsil, and asked that our patient be taken to an operating theatre despite the reluctance expressed by the emergency theatre team. Our patient’s hemoglobin immediately before being anaesthetized was 10.5g/dL, and his coagulation profile only showed an increased fibrinogen value, as expected from his continuing bleeding (Figure 1) but was otherwise normal. Intra-operatively, the blood trickling was not controlled after two attempts at superficial cautery by bipolar diathermy, and a right tonsillectomy with meticulous hemostasis was performed. Our patient was discharged the next day. The histology of the excised tonsil was suggestive of a benign non-specific ulcer on a background of chronic non-specific tonsillitis, with enlargement of the germinal centers. There was no evidence of vascular malformation, vasculitis, or fibrin thrombi formation.

## Conclusions

Spontaneous tonsillar hemorrhage has been associated with various pathological conditions. These primarily include acute or chronic tonsillitis, but may also involve peri-tonsillar or parapharyngeal abscesses, infectious mononucleosis, carotid aneurysm or pseudoaneurysm, and tonsillar cancer [3]. It is possible that the increased blood flow in the inflamed tonsillar tissue and the necrosis or trauma of the congested tonsillar vessels, or the extravasation of red blood cells from the engorged tonsillar vasculature, may lead to diffuse parenchymal bleeding [4,5].

**Figure 2:**
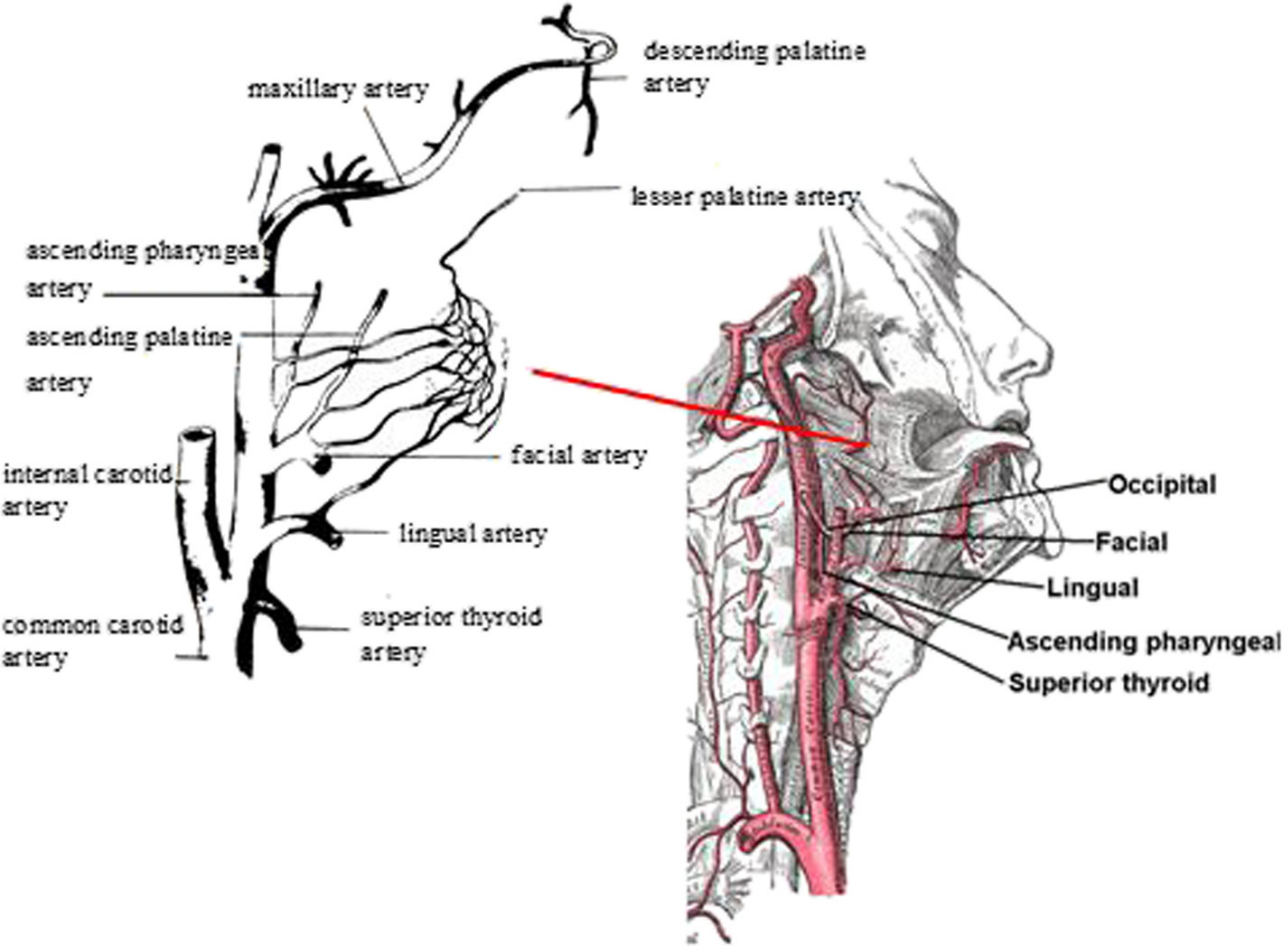
The blood supply to the palatine tonsil (modified from Wikipedia).

The palatine tonsil lies in a triangular space, bounded by the glossopalatine (anterior) and pharyngopalatine (posterior) arches. The blood supply of the tonsil comes from the tonsillar branch of the facial artery (main supply inferior pole), the tonsillar branches of the ascending palatine branch of the facial artery and the ascending pharyngeal branch of external carotid artery (posteriorly), the tonsillar branch of the dorsal lingual branch of the lingual artery (anteriorly), and the lesser palatine artery from the descending palatine branch of the maxillary artery (superior pole) (Figure 2). All these branches approach the tonsil from behind (that is, underneath its body), with the exception of the dorsal lingual branch, which penetrates the tonsil anteriorly, but not from the medial (visible) border.

Therefore it is evident that even though reports in the literature suggest attempting conservative local control of the bleeding initially, either with adrenaline soaked pledgets or cautery with silver nitrate [4,6], such attempts would be unsuccessful in definitively controlling a spontaneous tonsillar hemorrhage, as the source of bleeding requires removal of the tonsil to be accessed.

Unfortunately, vascular malformations ( i.e. carotid pseudoaneurysm) cannot be excluded in a patient that presents with spontaneous tonsillar hemorrhage, and can be an understandable cause of concern for the surgeon in view of performing an emergency tonsillectomy. Such malformations may be identified by performing a neck ultrasound, whereas an arteriogram can depict major vessel erosion in cases highly suspicious for cancer [3]. Regardless, a neck ultrasound is not always readily available, and prolonged invasive investigations may not be easily performed in an emergency setting in cases of considerable bleeding [1].

Hence, it seems more appropriate to perform one of the most common ENT procedures as first-line treatment for a potentially life-threatening complication of tonsillitis in an otherwise healthy individual, rather than risk the hemodynamic stability of a physically exhausted, and understandably terrified patient, stubbornly fiddling with pledgets and silver nitrate sticks.

## Consent

Written informed consent was obtained from the patient for publication of this case report and any accompanying images. A copy of the written consent is available for review by the Editor-in-Chief of this journal.

## Abbreviations

ENT: Ear nose and throat; GP: General practitioner; SHO: Senior house officer.

## Competing interests

The authors declare that they have no competing interests.

## Authors’ contributions

PVV operated on our patient, and wrote the manuscript. EI was a major contributor in writing the manuscript. Both authors read and approved the final manuscript.
